# Crocin Improves Damage Induced by Nicotine on A Number of Reproductive Parameters in Male Mice 

**DOI:** 10.22074/ijfs.2016.4771

**Published:** 2016-04-05

**Authors:** Mohammad Reza Salahshoor, Mozafar Khazaei, Cyrus Jalili, Mona Keivan

**Affiliations:** Fertility and Infertility Research Center, Kermanshah University of Medical Sciences, Kermanshah, Iran

**Keywords:** Crocin, Nicotine, Damage, Reproductive

## Abstract

**Background:**

Crocin, a carotenoid isolated from Crocus sativus L. (saffron), is a pharmacologically active component of saffron. Nicotine consumption can decrease fertility in males through induction of oxidative stress and DNA damage. The aim of this study is to determine the effects of crocin on reproductive parameter damages in male mice exposed to nicotine.

**Materials and Methods:**

In this experimental study, we divided 48 mice into 8 groups (n=6 per group): control (normal saline), nicotine (2.5 mg/kg), crocin (12.5, 25 and 50 mg/kg) and crocin (12.5, 25 and 50 mg/kg)+nicotine (2.5 mg/kg). Mice received once daily intraperitoneal injections of crocin, nicotine and crocin+nicotine for 4 weeks. Sperm parameters (count, motility, and viability), testis weight, seminiferous tube diameters, testosterone, and serum nitric oxide levels were analyzed and compared.

**Results:**

Nicotine administration significantly decreased testosterone level; sperm count, viability, and motility; testis weight and seminiferous tubule diameters compared to the control group (P<0.05). However, increasing the dose of crocin in the crocin and crocin+nicotine groups significantly boosted sperm motility and viability; seminiferous tubule diameters; testis weight; and testosterone levels in all groups compared to the nicotine group (P<0.05).

**Conclusion:**

Crocin improves nicotine-induced adverse effects on reproductive parameters in male mice.

## Introduction

A large, increasing number of patients worldwide use medicinal plants and herbs for health purposes (1). Crocin is a carotenoid obtained commercially from the dried trifid stigma of the culinary spice Crocus sativus L. (saffron) and is responsible for the red color of saffron (2,3). It is the diester formed from the disaccharide gentiobiose and dicarboxylic acid crocetin (4). Saffron has been used traditionally as a coloring or flavoring agent, as well as an herbal remedy (5). In traditional medicine, throughout history, saffron has been used to treat infertility, impotence, and as a sexual potential stimulant (6). It contains four major bioactive constituents: crocin (color), crocetin (color), picrocrocin (taste), and safranal (aroma) (7). 

Crocin can be purely isolated from the saffron extract and directly crystallized (8). The saffron spice contains numerous chemical substances such as carbohydrates, minerals, mucilage, vitamins (especially riboflavin and thiamin), and pigments that include crocin, anthocyanin, carotene, lycopene, and zigzantin (9,10). Crocin has also shown various pharmacological activities antioxidant, anti-tumor, radical scavenging, and genoprotective (11,13). The anti-tumor functions of crocin have a special place in pharmaceutics (14). According to research at pharmacological and high doses, crocin did not exhibit marked damages to all major organs of the body and no mortality was seen by crocin in mice (15). 

Infertility is a health problem that causes adverse effects in personal, social, and economic domains and is observed in 10 to 15% of couples (16). Approximately 40% of infertility problems are associated with males (17). Infertility in males has been associated with sperm dysfunctions such as low sperm count, immaturity, abnormality, and lack of motility (18). 

Various studies have shown that consumption of nicotine-containing compounds decreases sperm count and motility (19). Nicotine is a highly toxic organic compound that contains nitrogen and alkaloids which are mostly found in tobacco (20). 

Nicotine can easily pass through the cell membrane and react with tubulin protein present in the cytoplasm of multiplying cells, causing disorders to cell division (21). Nicotine can damage sperm membrane and DNA, and induce apoptosis in interstitial cells in the testis (22). We have taken into consideration the effect of saffron on hormone synthetics such as testosterone, the role of this hormone on spermatogenesis (23), the importance of the male reproductive system and lack of any report of a protective effect of crocin against nicotine in designing this study. Hence, the current study was conducted to analyze the protective effect of crocin on the damage induced by nicotine in a number of reproductive parameters in male mice. 

## Materials and Methods

### Chemicals

In this experimental study, digentiobiosyl-8, 8’-diapocarotene-8, 8’-oate (C_44_H_64_O_24_, crocin) powder (Merck, Germany) was diluted with normal saline (0.9%) to prepare the different doses. S)-3-[1-Methylpyrrolidin-2-yl]pyridine (C10H14N2, nicotine) solution (Merck, Germany) was diluted with normal saline (0.9%) prior to administration (24). 

### Animals

A total of 48 healthy adult Balb/c male mice that weighed 27-30 g were purchased from Tehran Razi Institute. Animals were kept at 22 ± 2ºC under controlled environmental conditions, 12/12 hour light/dark cycle and free access to water and food. Animals were maintained in compliance with National Institutes of Health guidelines (25). This study was conducted in accordance with the approval granted by the Ethical Committee for Research on Laboratory Animals at Kermanshah University. 

### Experimental design

The mice were randomly divided into 8 groups (n=6): i. Control (normal saline; 1 ml distilled water (DW)/daily), ii. Nicotine (2.5 mg/kg) (26), iii. Nicotine+crocin (12.5 mg/kg), iv. Nicotine+crocin (25 mg/kg), v. Nicotine+crocin (50 mg/kg), vi. Crocin (12.5 mg/kg), vii. Crocin (25 mg/kg), and viii. Crocin (50 mg/kg). Mice received intraperitoneal (IP) injections of nicotine once per day for 4 weeks. Crocin and nicotine+crocin were administered in the same way to the animals (24). 

### Testis weight and seminiferous tubule diameter

The testes were carefully removed, washed in normal saline solution (0.9%), blotted, and weighed separately. The average weights were used. After testes fixation by formalin, the histological process that included dehydration, clearing, and embedding was carried out. The microscopic sections (5 µm) were prepared for hematoxylin and eosin (H&E) staining. The seminiferous tubule diameters were measured by a Motic camera and software (Moticam 2000, Spain). Seminiferous tubule average diameter (µm) was determined for each testis (27). 

### Sperm collection

The cauda epididymis was excised, minced and incubated in a pre-warmed petri dish that contained 10 ml Hank’s balanced salt solution at 37°C. The spermatozoa were allowed to disperse into the buffer. After 20 minutes, the cauda of the epididymides were removed and the suspension was gently shaken to homogenize. The solution was analyzed under light microscope at a magnification of ×400 (22,28). 

### Sperm parameters 

In order to count the sperm, we diluted 500 μL of the sperm suspension with formaldehyde fixative [10% formalin in phosphate buffered saline (PBS)] (Sigma, USA). Approximately 10 μL from the diluted solution was transferred into a hemocytometer using a Pasteur pipette (Thoma, Assistant Sondheim/Rhön, Germany) and the solution was allowed to remain for 7 minutes. Then, the sperm that settled were counted and evaluated per 250 small squares of a hemocytometer (27). Viability was assessed by eosin Y staining (5% in saline). We placed 40 μL samples of the freshly prepared sperm suspension on a glass slide. The suspension was mixed with 10 μL eosin (Sigma, USA) and subsequently observed under a light microscope at ×400 magnification. Live sperm remained unstained whereas sperm that showed any pink or red coloration were classified as dead. At least 200 sperm were counted from each sample in 10 random fields of vision and we recorded the percentages of live sperm (29). In order to assess the percentage of motile sperm, the suspension was prepared by pipetting. A small aliquot (40 μL) of freshly liquefied semen was placed on a glass slide at 37˚C for film recording with a video microscope (Olympus, BX51, Germany). Randomly, we recorded 10 fields from each slide with a camera for sperm motility assessment via analysis of the recorded films. Sperm motility was divided into four levels according to certain criteria: i. Quick progressive motility in direct line, ii. Slow progressive motility in direct or indirect line, iii. No progressive motility, and iv. No motility (18). 

### Testosterone and nitric oxide levels

The animals were anesthetized 24 hours after the last injection. Blood was taken from the hearts of the animals and preserved at 37ºC for 30 minutes, then centrifuged (1000 g) for 15 minutes. The collected blood was centrifuged at 25°C and 4000 rpm for 10 minutes in order to obtain the serum. The serum samples were kept frozen at -18°C. The blood testosterone level was analyzed by enzyme linked immunosorbent assay (ELISA, Abcam 108666, USA). Nitric oxide was measured based on Griess colorimetric assay. Accordingly, N-(1naphthyl) ethylenediamine dihydrochoride (NEED), sulfonamide solutions and nitrite standards were prepared. To measure nitrite concentration in serum, the serum samples were thawed and 100 µl of the serum sample was deproteinized by zinc sulfate, then transferred to the wells. Subsequently, we added 100 µl chloride vanadium, 50 µl sulfonamide, and 50 µl NEED. The cells were incubated at 30°C in the dark. Optical densities (OD) of the samples were measured by an ELISA reader at a wavelength of 540 nm (30). 

### Statistical analysis

Data were presented as mean ± SEM and analyzed by one-way ANOVA followed by Tukey tests using SPSS package (version 18, SPSS Inc, USA). The Kruskal Wallis test was used to examine data normality and homogeneity of variances, considering a significance level of 0.05. 

## Results

### Testis weight and seminiferous tubule diameter

**Fig 1:**
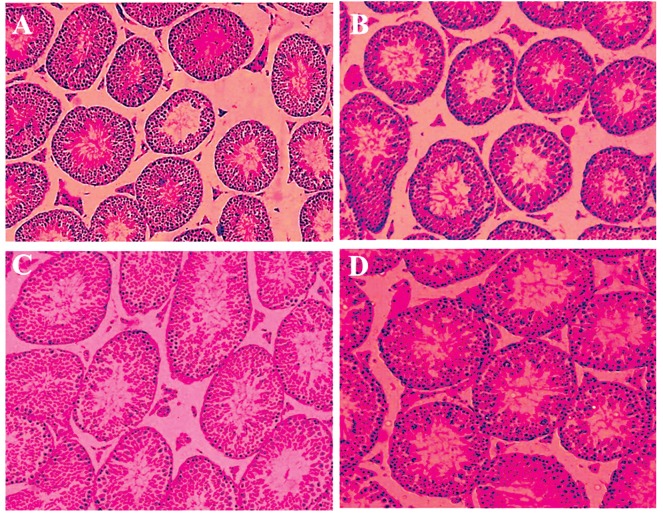
Effects of different concentrations of crocin on the diameters of seminiferous tubules according to hematoxylin and eosin (H&E) staining. A. Cross-section from the testis of mice from the control group with normal seminiferous tubules. Cross-sections from the testes of rats that received, B. 12.5 mg/kg of crocin, C. 25 mg/kg of crocin and D. 50 mg/kg of crocin (magnification: ×40).

The effective doses of nicotine (2.5 mg/kg) and crocin+nicotine (12.5 mg/kg) caused a significant decrease in testis weight and seminiferous tubule diameters compared to the control (saline) group (P=0.00). Crocin improved testis weight and seminiferous tubule diameters in treated animals of all doses compared with the nicotine group (P=0.00). Crocin+nicotine caused a significant increase in testis weight and seminiferous tubule diameters in all treated groups compared with the nicotine group. Crocin prevented the damage by nicotine on testis weight (P=0.00, Table 1, Fig .1).

### Sperm parameters

Mean sperm count, progressive motility, and viability significantly decreased in the nicotine (2.5 mg/kg) and crocin+nicotine groups of all doses compared to the control (saline) group (P=0.00). However, crocin and crocin+nicotine significantly improved high motility and sperm viability in all treated groups compared with the nicotine group (P=0.01). Crocin significantly improved sperm counts in all treated groups compared with the nicotine administered group (P=0.01). Increasing crocin+nicotine doses revealed no significant increase in the sperm count in the treated groups compared to the nicotine group (P=0.35). Crocin prevented the damage caused by nicotine on sperm parameters (Table 2).

**Table 1:** Effects of nicotine, crocin and crocin+nicotine on mean testis weight and diameter of seminiferous tubules in male mice (n=6 for each group)


Groups	Mean testis weight (g)	Diameter of seminiferous tubules (µm)

Control	0.12 ± 0.007^a^	46.12 ± 1.4^a^
Nicotine	0.065 ± 0.01^b^	25.39 ± 0.7^b^
Crocin 12.5 mg/kg	0.12 ± 0.003^ac^	44.25 ± 2.84^ac^
Crocin 25 mg/kg	0.13 ± 0.003^d^	46.99 ± 1.4^ac^
Crocin 50 mg/kg	0.13 ± 0.003^d^	50.87 ± 3.5^ad^
Crocin+nicotine (12.5 mg/kg)	0.083 ± 0.01^e^	34.79 ± 3.9^f^
Crocin+nicotine (25 mg/kg)	0.1 ± 0.003^f^	36.5 ± 0.8^f^
Crocin+nicotine (50 mg/kg)	0.1 ± 0.003^f^	37.88 ± 2.8^f^


Data are presented as mean±SEM. Values with different letters indicating significant differences among groups at P<0.05.

### Testosterone hormone and nitric oxide

**Fig.2:**
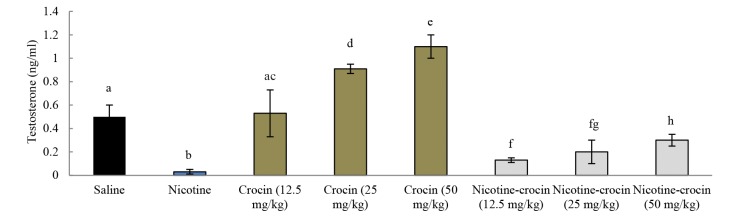
Effects of nicotine, crocin, and crocin+nicotine on testosterone levels in male mice (n=6 for each group). Different letters indicate significant differences among groups at P=0.00.

**Fig 3:**
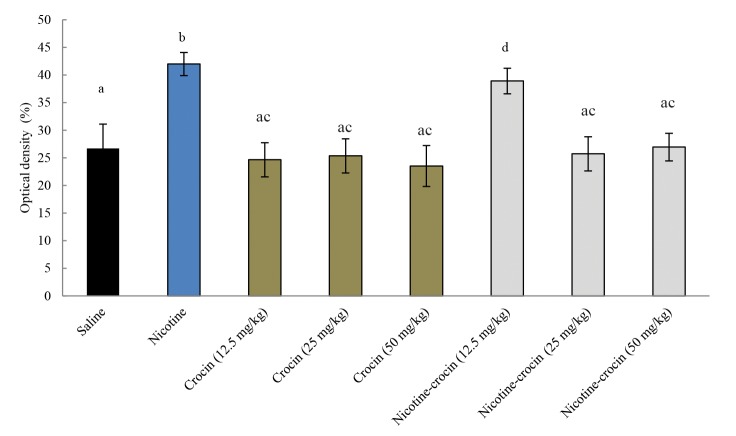
Effects of nicotine, crocin, and crocin+nicotine on mean nitric oxide levels in blood serum in male mice (n=6 for each group). Different letters indicate significant differences among groups at P=0.00.

Nicotine (2.5 mg/kg) and crocin+nicotine (12.5, 25 and 50 mg/kg) caused a significant decrease in testosterone compared to the control group (P=0.00). Increasing doses of crocin and crocin+nicotine showed significantly increased testosterone in all groups compared to the nicotine group. Crocin prevented the damage caused by nicotine on testosterone level (P=0.00, Fig .2). The mean level of nitric oxide in blood serum increased significantly in the nicotine (2.5 mg/kg) and crocin+nicotine (12.5 mg/kg) groups compared to the control group (P=0.00, Fig .3).

**Table 2:** Effects of nicotine, crocin and crocin+nicotine on sperm parameters in male mice (n=6 for each group)

Groups	Mean sperm count (10^6^)	Sperm progressive motility (%)	Sperm viability (%)
Control	4.53±0.06^a^	6.6±0.16^a^	77.83±0.16^a^
Nicotine	2.16±0.5^b^	0.02±0.05^b^	30.03±0.05^b^
Crocin1 2.5 mg/kg	4.5±0.43^ac^	6.83±0.9^a^	85.06±0.9^c^
Crocin 25 mg/kg	4.52±0.7^ac^	8.83±1.04^ac^	85.75±1.04^c^
Crocin 50 mg/kg	4.69±1^ac^	11.83±3.07^d^	89.45±3.07^c^
Crocin+nicotine (12.5 mg/kg)	2.16±0.5^bd^	0.3±0.08^e^	59.25±0.08^d^
Crocin+nicotine (25 mg/kg)	2.6±0.7^e^	1.3±0.9^f^	63.95±0.9^e^
Crocin+nicotine (50 mg/kg)	2.9±0.2^f^	1.5±0.5^f^	70.61±0.5^f^

Data are presented as mean ± SEM. Values with different letters indicating significant differences among groups at P<0.05.

## Discussion

Currently, medicinal plants have numerous applications. One of the target tissues for plant extracts is reproductive organs such as the testis and reproductive parameters. The results of our experimental study have revealed that nicotine promoted male reproductive toxicity in mice. This agreed with results by Sankako et al. (31) who reported residual damage on sperm concentration, motility, and morphology after cigarette smoke exposure.

In fertile individuals, sperm motility levels have a direct relation to fertilization ability (32). Crocin has possibly increased the count and motility of normal sperm in treated groups through enhancing the antioxidant defense of the body (3). On the other hand, crocin caused a significant change in reproductive indices and inhibited the harmful effects induced by nicotine in the reproductive hormone. Crocin could act as an antioxidant and improve the sperm quality by increasing the expression of antioxidant genes in comparison with the nicotine group (11).

The findings of this study were in line with the results of a study conducted by Kalpana et al. (33) that investigated relative peroxidative and antioxidant effects of curcumin on nicotine-induced toxic fatty tissue. They reported that curcumin (as an antioxidant) decreased the toxicity induced by nicotine in fatty tissue. Researchers have stated that increasing free radicals causes the loss of epithelial cells, which can destroy cytoplasmic bridges and consequently decrease sperm count and motility levels, increasing sperm malformation (34).

Antioxidant properties of crocin can improve sperm quality by increasing expression of antioxidant genes (13). Nicotine can directly inhibit primary Leydig cell testosterone levels, but the mechanism of this effect is not known. Nicotine leads to lower testosterone hormone production, which may be a secondary reason for reduction of sperm number in seminiferous tubules (35). Changes in sperm vitality and motility after nicotine injection may be due to an increase in reactive oxygen species (ROS) levels in mice semen. Several lines of evidence indicate that ROS is involved in nicotineinduced testicular damage (36).

The results showed that sperm count, motility, and viability in the presence of crocin significantly improved compared to nicotine-only-treated animals. Therefore, positive changes in the sperm quality might be due to the hydroxyl radical scavenging activity of crocin which has been shown to inhibit lipid peroxidation (11). Increased sperm counts might possibility be caused by the anti-apoptotic effects of crocin (9). Crocin has been shown to act like an anti-oxidant *in vivo*, preventing the formation of free radicals and lipid peroxidation, hence, preventing oxidant-induced apoptosis (12). The results of the present study have confirmed findings reported by Asadi et al. (6) which indicated that saffron improved epididymal sperm parameters in rats that were exposed to cadmium.

Crocin may reduce hypophyseal-hypothalamus sensitivity to testosterone feedback control on luteinizing hormone (LH) secretion. In light of crocin antioxidants’ effects in biosynthesis of steroid hormones, it seems that crocin can affect male sexual hormone concentrations (37). Crocin administration improves sperm parameters and most changes that occur on testicle tis-sue in mice probably are the result of increasing testosterone levels. The findings of the present study have confirmed the results by Khayatnouri et al. (23) where saffron administration improved the spermatogenesis index in rats. However, the results of this study contrasted the findings of Safarinejad et al. (38) who reported that saffron administration for 26 weeks to infertile men with idiopathic oligoasthenoteratozoospermia (OAT) had no effects on semen parameters.

In the current study, the mean nitric oxide in blood serum has increased significantly in the nicotine group compared to the control group. Nitric oxide and the signal pathways of 3', 5'-cyclic guanosine monophosphate (cGMP), an important cascade signal, are found in many mammalian cells such as Sertoli cells and germinal cells in the testis tissue (39). Nitric oxide plays a pivotal role in blood circulation regulation in the reproductive system and previous studies have reported an increase in nitric oxide expression along with apoptosis in germinal cells (40).

## Conclusion

The findings of this study showed that crocin improved some of the reproductive parameters in mice treated with nicotine. The antioxidant effects of crocin might have been a major reason for its positive impact on reproductive parameters. However, further studies are required to define its exact mechanism of action.
